# Consideration of Abscesses in Mouths of Children

**Published:** 1922-07

**Authors:** F. W. Hollister

**Affiliations:** 704 Journal Building, Portland, Oregon


### CONSIDERATION OF ABSCESSES IN MOUTHS OF CHILDREN

BY F. W. HOLLISTER, D.M.D., 704 JOURNAL BUILDING, PORTLAND, OREGON

There are a great many things to be considered before we decide, definitely, whether to attempt the treatment of abscessed conditions or extract the teeth that cause them.

A few simple rules which I have sort of formulated to enable me to diagnose the future of an abscessed tooth which comes under my care, are:

First of all, get a good radiograph of the case, and study it well.

Look well into the general condition of the child.

The condition of the abscess itself, as to duration, extent of involvement, etc.

If a deciduous tooth, the length of time it should be retained to permit its successor to properly erupt.

If a permanent tooth, the necessity of saving it to insure proper occlusion (for example the six-year molars).

Or to be of importance to be used in the attachments for regulating appliances, and various other reasons which might arise.

Personally, unless in extreme cases similar to some of the aforementioned, I would not advise attempting the treatment of chronic abscessed conditions where there be a fistula with a great deal of destruction of surrounding tissue. When the radiograph shows this condition to unquestionably exist, my advice is the removal of the tooth causing same, and the proper cleaning up of the area involved, unless, as before mentioned, the condition of the patient warranted it and it be of vital importance to save the tooth for a short while only. But *never* in these cases would I advise treatment with the idea or understanding that it was to terminate as a permanent matter.

In substance allow me to state. If you find abscessed conditions in a child's mouth, and you feel justified in attempting the treatment of them, all well and good—let your conscience be your guide. But should you not feel justified in treating, then do the next best thing or perhaps the best thing: extract the tooth or teeth that are causing the trouble. At any extreme, rid the child's system of the pus.

If my memory serves me right, I believe that Dr. Weston A. Price is the author of a statement that pus from an abscessed deciduous tooth with a fistulous opening is sterile pus. This I am not ready to accept as a set rule to go by, as I believe that we all have come in contact with pus from fistulous abscessed deciduous teeth that we felt was anything but sterile.

If you will permit me to deviate a little from the subject assigned, there is one little point I should like very much to bring out. In cases where we find it absolutely necessary to extract deciduous teeth some little time before their successors are due to rupt, *always* make it a point to see that some sort of a retaining appliance be put on to retain the space that nature provided those little teeth to keep until time for the permanent ones to take their places.—*Northwestern Journal.*

